# Localized social capital in action: How neighborhood relations buffered the negative impact of COVID-19 on subjective well-being and trust

**DOI:** 10.1016/j.ssmph.2022.101307

**Published:** 2022-12-05

**Authors:** Christoph Zangger

**Affiliations:** University of Bern, Institute of Sociology, Fabrisktrasse 8, 3012, Bern, Switzerland

**Keywords:** Neighborhood effects, Neighborhood relations, Subjective well-being, Trust, COVID-19, Panel data

## Abstract

The importance of neighbors is highlighted in times of crisis, such as the COVID-19 pandemic: they offer support by providing small services and a sense of community. Using panel data from Switzerland, this study investigates how and for whom relations with neighbors changed during the pandemic. In a second step, changes in subjective well-being and trust in other people are linked to changes in neighborly relations. The results show that the negative impact of the pandemic on people’s subjective well-being and trust was much less pronounced for those who improved their relations with neighbors. Meanwhile, people with more resources prior to the pandemic were generally more likely to improve neighborly relations. Consequently, the most vulnerable groups in terms of health and socio-economic status saw their subjective well-being and trust negatively impacted through the challenging circumstances of the pandemic as well as indirectly through a relative deterioration of neighborly relations. Robustness analyses further show that the documented effects are attributable to changes induced by the pandemic.

## Highlights

• Improving neighborly relations during COVID-19 buffered the negative effect on subjective well-being and trust.
• Geographically more disperse social networks such as friends or online networks are of subordinate importance.
• Meanwhile, people with more resources were more likely to improve relations with neighbors.
• Robustness analyses show that the effects can be attributed to the unique circumstances of the pandemic.

## 1 Introduction

Apart from friends and family, neighbors are among the first to provide help and support (LaLone, 2012; Wellman & Wortley, 1990; Zetterberg, Santosa, Ng, Karlsson, & Eriksson, 2021). More generally, neighborhoods and localized social networks are crucially associated with people’s well-being (Ludwig et al., 2012; Sampson, Morenoff, & Gannon-Rowley, 2002). While much research has focused on neighborhood effects on outcomes such as educational chances, labor market participation, or physical health (Brattbakk & Wessel, 2013; Oakes, Andrade, Biyoow, & Cowan, 2015; Sampson et al., 2002; Zangger, 2019), researchers increasingly also investigate the effects of neighborhood environments and networks on people’s subjective well-being (Bonomi Bezzo, Silva, & van Ham, 2021; Farrell, Aubry, & Coulombe, 2004; Jaramillo, Rohe, & Webb, 2020). Following Diener (2009), subjective well-being comprises an overall assessment of one’s satisfaction with life as well as of pleasant and unpleasant experiences (positive and negative affect). Given its association with people’s health (Dong & Qin, 2017; Jaramillo et al., 2020), subjective well-being can also be considered an additional path through which neighborhoods and neighbors affect individual life chances and health-related outcomes.

Recently, many people’s subjective well-being has decreased significantly during the COVID-19 pandemic and in response to the corresponding social confinement measures (e.g., stay-at-home orders) (Bonomi Bezzo et al., 2021; Zacher & Rudolph, 2021). Likewise, the pandemic also led to increasing tensions and an erosion of trust (Kye & Hwang, 2020; Sibley et al., 2020). At the same time, we witnessed an increase in neighborhood help initiatives, for example to organize grocery shopping and other small services for at-risk groups (Miao, Zeng, & Shi, 2021; Zetterberg et al., 2021). Against this background, the present contribution investigates to what extent neighborhood relations changed in the course of the COVID-19 pandemic and whether neighborhood networks buffered the negative effects of the pandemic on subjective well-being and trust.

This paper pursues two goals: first, using longitudinal data from Switzerland, it examines how and for whom neighborhood relations changed during the pandemic. Second, and as the first study of its kind, it relates changes in neighborly relations to changes in subjective well-being and generalized trust. Focusing on subjective well-being and generalized trust not only follows from the crucial role of social capital (Putnam, 2000; Zetterberg et al., 2021), but it also allows for an assessment of both individual (subjective well-being) and societal consequences (trust) of neighborhood relations in times of crisis and increased strain. In this regard, the unforeseeable changes and restrictions in people’s social lives during the pandemic provide a unique opportunity to study the exogenous effects of neighborhood social capital on individual subjective well-being and trust.

## 2 Background

### 2.1 Neighborhoods, neighbors, subjective well-being, and trust

Apart from physical aspects of the neighborhood (e.g., public green spaces; e.g., Dong & Qin, 2017; Jaramillo et al., 2020), neighborhood composition is discussed as a prominent explanation for how neighborhoods affect individual subjective well-being (Farrell et al., 2004; Ludwig et al., 2012). In this regard, localized social capital and the therein mobilized resources are put forward as a mechanism (Cramm, van Dijk, & Nieboer, 2013; Farrell et al., 2004; Helliwell & Putnam, 2004; Howley, Neill, & Atkinson, 2015). People in one’s vicinity can provide resources and help (Aldrich & Meyer, 2015; LaLone, 2012), which stresses the importance of neighbors with more resources (Seifert & König, 2019). On the one hand, supporting one’s neighbors requires time, for example to go shopping for people with reduced mobility. On the other hand, neighbors with higher educational degrees and income can be an advantage when one is need of help with official documents and processes or for small favors. In line with this argument, existing research finds a positive association of individual subjective well-being and neighbors’ income (Kingdon & Knight, 2007; Ludwig et al., 2012). This effect, however, is contrasted by a negative effect of the median income at the regional level (Ifcher, Zarghamee, & Graham, 2018). The complex pattern then indicates a second mechanism of compositional effects on subjective well-being: people compare themselves with others, which can result in relative deprivation effects (Howley et al., 2015).

Third, neighborhood social capital more generally comes along with social cohesion and trust among neighbors (Middleton, Murie, & Groves, 2005; Perkins, Subramanian, & Christakis, 2015). Interactions with neighbors result in an increased sense of community and mutual support, and – especially in difficult times – lead to social connections that protect against sadness, loneliness and low self-esteem (Helliwell & Putnam, 2004). Empirical evidence from a variety of contexts supports this assumption (Cramm et al., 2013; Helliwell & Putnam, 2004; Howley et al., 2015). Of note, Greenfield and Reyes (2015) show that especially changes in the relation with one’s neighbors are associated with subjective well-being.

While the outlined mechanisms address subjective well-being, much of the reasoning can also be applied to trust. Localized social capital and a sense of community support the formation of trust at the local as well as the wider, societal level (Delhey, Newton, & Welzel, 2011; Hays, 2015). Consequently, changes in neighborly relations and neighboring likely also affect trust in other people. Moreover, studies point to the importance of neighborhood composition (e.g., Gereke, Schaub, & Baldassarri, 2018), although this effect is again dependent on the amount of interaction with neighbors (Stolle, Soroka, & Johnston, 2008). Neighborhood networks and localized social capital are thus also important factors for building trust in other people.

### 2.2 Neighborhood networks in times of COVID-19

The importance of localized social networks is emphasized in times of crisis: neighbors provide shelter and help during natural disasters (Aldrich & Meyer, 2015; LaLone, 2012) and they support people suffering from mental distress (Dong & Qin, 2017; Greenfield, 2016). This also holds true for the COVID-19 pandemic (Miao et al., 2021; Zetterberg et al., 2021): neighborhood help initiatives were essential in providing support to at-risk groups, especially when social confinement measures were in place.

Meanwhile, the pandemic and the measures to combat its spread have had a significant effect on people’s subjective well-being, social cohesion, and trust (Carbone, 2020; Devine, Gaskell, Jennings, & Stoker, 2020; Sibley et al., 2020). Studies from around the world have shown that subjective well-being decreased during local and national lockdowns (Sibley et al., 2020; Zacher & Rudolph, 2021). Similar effects are also reported for people’s generalized trust (Kye & Hwang, 2020; Thoresen, Blix, Wentzel-Larsen, & Birkeland, 2021).

However, existing studies show that the negative effects of the pandemic on subjective well-being and trust differ by social class, education, political ideology, and contact with people who tested positive for COVID-19 (Kye & Hwang, 2020; Thoresen et al., 2021; Zacher & Rudolph, 2021). Moreover, using longitudinal data from the UK, Bonomi and colleagues (2021) find that the decrease in subjective well-being was particularly pronounced in more deprived areas. This finding is further supported by Miao et al. (2021) who show that the adverse effect on people’s mental health depended on the amount of services provided by local voluntary associations.

The last two studies then suggest an additional mechanism: with social distancing and lockdown measures in place, help from and contact with neighbors became an especially important source for coping with the pandemic. However, the change in neighborly relations during that time depends, as Zetterberg et al. (2021) show, on the amount of pre-pandemic localized social capital.

Against this background, the following hypotheses are proposed. First, people with a larger neighborhood network prior to the pandemic – in terms of contact and the exchange of mutual support – should experience greater improvements in neighborly relations. This can be attributed to a stronger involvement in neighborhood-based coping strategies, such as going shopping for at-risk neighbors, which requires both time and money. Although people with less resources benefit most from neighborhood networks (De Meulenaere, Baccarne, Courtois, & Ponnet, 2020), those with more advantageous endowments are found to mobilize more support through such networks (Seifert & König, 2019). In other words: giving support and being able to do so should result in greater improvements in neighborly relations than receiving support. Compared to those in need of help during COVID-19 (i.e., at-risk groups), people who had the time and money to help were more likely to meet more and new people in their surroundings.

Second, locally organized support was especially important during the pandemic (De Meulenaere et al., 2020; Miao et al., 2021; Zetterberg et al., 2021). With stay-at-home orders in place in Switzerland and elsewhere, in-person contacts were limited to the vicinity of one’s home and neighbors thus became more important and more likely partners for interaction. Meanwhile, geographically more disperse networks of friends or online networks should be of subordinate importance.

Third, the decline in people’s subjective well-being and generalized trust will likely differ with the available resources to cope with the crisis (Banerjee, 2020; Devine et al., 2020). Although job-loss and financial anxieties due to the pandemic were less of an issue in Switzerland, an increase in neighboring activities and local solidarity should nevertheless strengthen community resilience trust (Aldrich & Meyer, 2015; LaLone, 2012; Zetterberg et al., 2021). Consequently, a COVID-19-induced increase in neighboring, understood as the exchange of social and instrumental support (Farrell et al., 2004), is expected to buffer the negative effects of the pandemic on people’s generalized trust and subjective well-being.

## 3 Materials and methods

### 3.1 Data

To test the hypotheses, this study uses data from the Swiss Household Panel (SHP; Tillmann et al. (2016)). The SHP is a representative annual, longitudinal study comprising somewhat more than 7’000 households and about 12’000 individuals. While data is usually collected at the end of each year, an additional data collection took place between 12th of May and June 26th^,^ 2020, right after the end of the first lockdown in Switzerland (Refle et al., 2020). 67% of all invited respondents from the previous wave (2019) completed a short 15 min, self-administered paper or online survey, which corresponds to 5’843 people from 4’053 households. The comparably lower response rate of the COVID-19 survey can be attributed to a change in survey mode (self-administered vs. face-to-face). Since there are no longitudinal weights available for the COVID-19 survey and because using the cross-sectional weights for that survey yield the same results, the following analyses are based on unweighted models.

The COVID-19 questionnaire focused on the effects of the pandemic on people’s economic and social life. Meanwhile, there are several measures that allow for a comparison with previous waves, including people’s subjective well-being and generalized trust. This study uses data from the COVID-19 survey and the previous wave of data, collected at the end of 2019 (wave 21). Restricting the sample to adults and omitting cases with missing values on any of the included variables resulted in 4’530 and 4’276 cases for the models concerned with subjective well-being and trust, respectively. Respondents in the final sample are between 18 and 99 years old, about one third holds a tertiary degree, and about one in 20 households moved since the last wave of data collection. Additional information about the sample can be found in Table A1 in the appendix.

#### 3.1.1 Measures

This study assesses subjective well-being by means of people’s rating of the overall satisfaction with life on an 11 point scale (Diener, Emmons, Larsen, & Griffin, 1985). Trust is measured through respondents’ assessment of “whether you cannot be too careful in your encounters with people or whether most people can be trusted” on an 11 point scale (Yamagishi & Yamagishi, 1994). Both measures are well-established and validated in the Swiss and especially the international context (e.g., Kuhn et al., 2021).

Of primary interest is whether changes in subjective well-being and generalized trust are associated with changes in people’s neighborhood network. In the SHP, social networks are inquired only every third year, the last time at the end of 2019. The COVID-19 questionnaire, however, asked to what extent respondents’ relation with their neighbors changed since the start of the pandemic (*Overall, has the relationship with your neighbors deteriorated or improved since the Corona crisis began?* – answers from 0, deteriorated a lot, to 10, improved a lot). Other social network measures were not part of the COVID-19 questionnaire. From the previous wave, however, information about people’s friendship, neighborhood, and online social networks is available (size, frequency of contact, and amount of support received). Likewise, whether respondents have a partner and/or children is used to assess their family network. All additional measures, if not stated otherwise, were collected in early summer 2020, that is, after the end of the first lockdown in Switzerland.

People’s subjective health (measured on a 5-point scale) and own or friends’ COVID-19 infections are used as health-related covariates. Furthermore, people’s age is included linearly as well as a quadratic term to account for the fact that the elderly were more affected by the pandemic and that they usually also rely more on instrumental help from neighbors (Seifert & König, 2019).

Additionally, all models account for people’s socioeconomic situation by means of their occupational status (employed vs. self-employed vs. economically inactive people who are unemployed, retired or otherwise not gainfully employed) and their highest education (at most compulsory schooling, upper secondary, and tertiary education). Moreover, the models control for households’ financial situation, differentiating between those who can save money, those who just get by, and those who get into debt.

All models further control for prior residential mobility and the Swiss Statistical Office’s community typology since existing research stresses that, compared to urban areas, the average subjective well-being is usually higher in rural areas and small towns (Farrell et al., 2004; Kingdon & Knight, 2007). The models further account for respondents’ gender and the duration of residence in Switzerland. Finally, neighborhood vandalism is included to control for differences in neighboring according to perceived neighborhood disorder (*Are you faced with any of the following problems concerning your accommodation? – Crime, violence or vandalism in the area*). Since religiosity and civic engagement have been found to be important determinants (Putnam, 2000), they are included when modeling generalized trust. Descriptive statistics of all measures can be found in Table A1 in the appendix.

### 3.2 Methodological approach

The analyses proceed in two steps. First, it is evaluated whose relations with neighbors changed to the better or worse. This is followed by an examination of the extent to which changes in neighborhood relations buffered the negative impacts of the pandemic on subjective well-being and generalized trust.

Although the data are longitudinal, the change in neighborhood relations was collected as perceived change compared to pre-COVID times. This asks for an analytical strategy that relates the perceived change to the repeated measurement of other variables. Change score analysis is a framework that enables an integration of these two perspectives (Allison, 1990; Morgan & Winship, 2014). For the first analytical step, we thus estimate the equation

(1) δY=Xβ+ϵ,

where *δ***Y** is a vector of the perceived difference in neighborhood relations and ***X*** is a *n* × *p* matrix of observed covariates with ***β*** the corresponding *p* × 1 coefficient vector. It is important to note that *δ***Y** reflects respondent’s self-assessed difference of how relations with neighbors changed rather than the difference of the same measure at two time points (i.e., for the first step of analysis *δ***Y** ≠ **Y**_*t*_−**Y**_*t*−1_). Most importantly, using a self-reported measure of change does not come with the advantage of controlling for unobserved heterogeneity by means of differing out the effects of time-constant unobserved influences (Morgan & Winship, 2014). In the first step of analysis, change in neighboring is analyzed as a categorical variable, differentiating between relations that deteriorated, stayed the same, or improved during the pandemic by means of a multinomial logit model. Average marginal effects are reported to allow an interpretation on the basis of probabilities rather than odds ratios.

In the second step, the longitudinal nature of the data is used more directly: people’s subjective well-being and generalized trust are measured in the interim COVID-19 questionnaire with the same items as in pre-COVID-19 waves. However, since the key independent variable, the change in relations with neighbors, is measured post-hoc as perceived change, random or fixed effects models are not an option. With random or fixed effects it would only be possible to assess the overall impact of COVID-19 on subjective well-being and trust, but not the effect of perceived changes in neighborly relations. Thus, a change score approach is again the alternative closest to the two approaches (Allison, 1990; Morgan & Winship, 2014).

However, using panel data from previous waves allows us to further investigate whether the results can truly be attributed the changes in neighboring caused by the pandemic. To this end, the models will be re-estimated using pre-COVID waves as a robustness test.

## 4 Results

### 4.1 Neighborhood relations in the pandemic

**Fig. 1:**
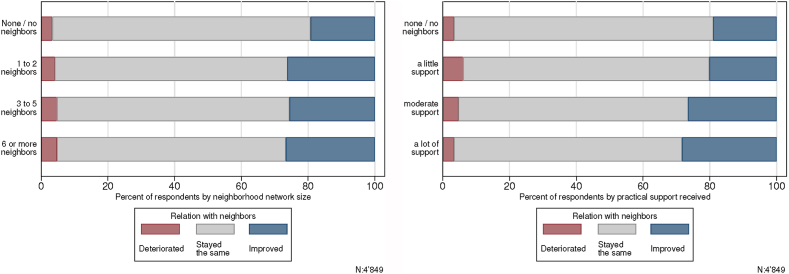
Change in neighborhood relations.

Starting with Fig. 1, we infer that about every 5th person reports an improvement in their relations with neighbors. In line with the expectation, respondents with a larger network of neighbors before the onset of COVID-19 are more likely to have experienced an improvement in their neighborly relations (Fig. 1a). This pattern is even more pronounced in the case of instrumental support received from neighbors (Fig. 1b). However, people who had received more instrumental support prior to the crisis are likely also more affected by the pandemic and social confinement, such as the elderly.

Moreover, unequal resources and opportunities for interaction should make a difference. For a more complete picture, Table 1 presents the results (as average marginal effects) of a multinomial logit model. Compared to those who had received no support from their neighbors prior to the pandemic, people who had received moderate or a lot of support have a 8.9 and 11 percentage points (pp) higher probability of improving the relation with their neighbors, respectively. Meanwhile, people with little support are also 3.2 pp more likely to report a deterioration of relations. Once the neighborhood network is accounted for, the effects of other social networks are mostly absent. Only respondents who had weekly contact with their friends and with a larger online social network prior to the pandemic are also more likely to report an improvement in neighboring.

**Table 1:** Change in neighborhood relation during the pandemic – AME.

	deteriorated		stayed the same		improved	
Support from neighbors (ref.: *no support/no neighbors*)						
a little support	0.032*	(0.014)	−0.072**	(0.027)	0.040	(0.025)
moderate support	0.012	(0.007)	−0.101***	(0.015)	0.089***	(0.014)
a lot of support	0.002	(0.008)	−0.112***	(0.019)	0.110***	(0.018)
Contact with friends (ref.: *no contact/no friends*)						
less then once a month	−0.024	(0.025)	0.009	(0.045)	0.014	(0.041)
monthly	−0.026	(0.021)	−0.036	(0.036)	0.062	(0.032)
weekly	−0.028	(0.020)	−0.050	(0.035)	0.077*	(0.031)
Log(size online social network)	−0.000	(0.000)	−0.002	(0.001)	0.002*	(0.001)
Health	−0.014***	(0.004)	0.012	(0.010)	0.002	(0.009)
COVID-19 infection (ref.: *no infection*)						
respondent or someone in household	0.040	(0.028)	−0.105*	(0.052)	0.064	(0.049)
someone respondent knows personally	0.000	(0.006)	−0.068***	(0.014)	0.067***	(0.013)
Household finances (ref.: *HH can save money*)						
HH spends what it earns	−0.009	(0.006)	−0.007	(0.015)	0.015	(0.014)
HH eats its assets or gets into debt	0.024	(0.013)	−0.058*	(0.024)	0.034	(0.023)
Education (ref.: *at most compulsory schooling*)						
upper secondary education	−0.002	(0.010)	−0.073***	(0.020)	0.076***	(0.018)
tertiary education	−0.002	(0.011)	−0.160***	(0.023)	0.161***	(0.021)
Gender (ref.: *man*)						
woman	−0.009	(0.006)	−0.042**	(0.013)	0.051***	(0.013)
Housing type (ref.: *(semi-)detached house*)						
apartment building	0.001	(0.006)	−0.044**	(0.014)	0.043***	(0.013)
other	−0.014	(0.013)	0.025	(0.033)	−0.011	(0.032)
Community typology (ref.: *urban centers*)						
suburban communes	−0.032*	(0.013)	0.079**	(0.026)	−0.047	(0.024)
wealthy communes	−0.021	(0.016)	0.050	(0.034)	−0.029	(0.032)
peripheral urban communes	−0.030]*	(0.014)	0.074**	(0.027)	−0.045	(0.026)
tourist communes	−0.034	(0.019)	0.102*	(0.042)	−0.068	(0.040)
industrial and tertiary sector communes	−0.024	(0.014)	0.080**	(0.029)	−0.056*	(0.027)
rural commuter communes	−0.021	(0.013)	0.035	(0.027)	−0.014	(0.025)
mixed agricultural communes	−0.033*	(0.014)	0.077**	(0.029)	−0.043	(0.028)
peripheral agricultural communes	−0.040*	(0.016)	0.058	(0.037)	−0.018	(0.036)
Observations	4’641					
Pseudo-R^2^	0.043					

Standard errors in parentheses; *p < 0.05, **p < 0.01, ***p < 0.001; Controlled for relationship status, children, household relocation, occupational status, age, and age^2^.

Furthermore, respondents in better health are less likely to report a deterioration of neighborly relations (by 1.4 pp). Likewise, people are less likely to report no change in their relations with neighbors if they or their friends went through a COVID-19 infection (by 10.5 and 6.8 pp, respectively). Resources are important as well: if respondents are in a tight financial situation, they are 5.8 pp less likely to report no change in their relations with neighbors. Meanwhile, people with secondary schooling have a 7.6 pp higher probability, and those with tertiary degrees have a 16.1 pp higher likelihood to report an improvement in neighboring compared to those who at most completed compulsory education.

Compared to men, women have a 5.1 pp higher probability to report an improvement in neighboring during the pandemic. People living in an apartment building rather than a single house are also 4.3 pp more likely to improve relations with neighbors. Finally, compared to people in urban centers, respondents in other regional structures are more likely to report no change in neighborly relations. All these effects are additionally controlled for age, age^2^, partnership status, presence of children, occupational status, and residential mobility.

### 4.2 Neighborhood relations, subjective well-being and trust in times of crisis

Table 2 depicts the second step of analysis, where changes in neighborly relations are used to explain changes in subjective well-being (first column) and trust (second column). Positive coefficients imply more positive change in subjective well-being and trust, that is, an improvement (or rather, less deterioration) compared to pre-COVID-19 times.

**Table 2:** Change in subjective well-being and generalized trust during the pandemic.

	2020 vs. 2019			
	Change score SWB		Change score trust	
Change neighborly relations	0.386***	(0.079)	0.278*	(0.125)
(Change neighborly relations)^2^	−0.029***	(0.006)	−0.018	(0.010)
Log(size neighborhood network)	0.007	(0.004)	−0.013*	(0.006)
Log(size friendship network)	−0.005	(0.008)	−0.019	(0.012)
Log(size online social network)	−0.004	(0.003)	−0.009	(0.005)
Days since end of lockdown	0.009***	(0.002)	0.003	(0.003)
Health	0.332***	(0.030)	0.050	(0.048)
COVID-19 infection (ref.: *no infection*)				
respondent or someone in household	−0.201	(0.154)	0.128	(0.241)
someone respondent knows personally	−0.067	(0.042)	0.045	(0.067)
Household finances (ref.: *HH can save money*)				
HH spends what it earns	0.213***	(0.047)	0.155*	(0.074)
HH eats its assets or gets into debt	0.227**	(0.072)	0.054	(0.113)
Gender (ref.: *man*)				
woman	−0.110**	(0.042)	−0.085	(0.067)
Community typology (ref.: *urban centers*)				
suburban communes	−0.108	(0.078)	−0.058	(0.123)
wealthy communes	−0.136	(0.102)	0.106	(0.162)
peripheral urban communes	−0.086	(0.083)	−0.054	(0.131)
tourist communes	−0.062	(0.133)	0.016	(0.212)
industrial and tertiary sector communes	−0.201*	(0.087)	0.060	(0.139)
rural commuter communes	−0.053	(0.079)	−0.029	(0.125)
mixed agricultural communes	−0.044	(0.088)	0.026	(0.139)
peripheral agricultural communes	−0.032	(0.113)	−0.100	(0.181)
Voluntary work			0.044	(0.067)
Feeling of religiosity			0.063*	(0.029)
Observations	4′338		4′111	
*R*^2^	0.060		0.016	

Standard errors in parentheses, Controlled for household relocation, occupational status, education, relationship status, children, duration of residence in Switzerland, neighborhood vandalism, community typology, age, and age^2^; *p < 0.05, **p < 0.01, ***p < 0.001.

**Fig. 2:**
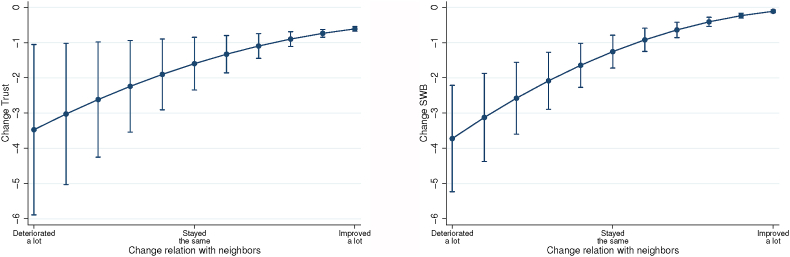
Change of subjective well-being and trust by change in neighborly relations.

Starting with subjective well-being, we note that people who improved their relations with neighbors during the pandemic also experienced more positive change in their subjective well-being. However, this effect is non-linear, indicated by the significant, negative quadratic term. People who improved their relations with neighbors a lot experienced only a minor decline in subjective well-being compared to pre-COVID-19 times (left panel of Fig. 2). This effect is independent of other networks, namely respondents’ friendship and online social network prior to the pandemic.

Respondents in better health are more likely to experience more positive (i.e., less negative) change in subjective well-being during the pandemic. Also, the more days have passed since the end of the lockdown, the more positive the change in subjective well-being. The rest of the control variables have only a subordinate or no influence. People with financial difficulties are more likely to have a positive change score. This might, however, merely reflect that they reported lover levels of well-being to begin with (floor effect). Compared to men, women experienced more negative changes in their subjective well-being in the course of the pandemic. Meanwhile, there is almost no difference in the change in subjective well-being among different regional structures. However, there were considerable regional differences in change of neighboring. One explanation might thus be that regional differences in subjective well-being work through differences in neighboring and housing.

The second column of Table 2 reveals a similar pattern for the change in generalized trust. Again, people who improved their relations with neighbors have a more positive change score, that is, their trust in others was less affected by the pandemic (see Fig. 2b). Unlike before, this effect is linear, and people who improved their relations with neighbors a lot nevertheless report a small decline in trust (Fig. 2). Even though Fig. 2b indicates a slight non-linearity, the generalized trust of people whose relation with neighbors deteriorated a lot is not as disproportionately affected as in the case of subjective well-being. In line with the expectation, most of the other network measures (e.g., friends, online network) have no additional effect.

The insignificant effect of the number of days since the end of the first lockdown suggests that the negative impact of the pandemic is longer lasting for generalized trust than for subjective well-being. Moreover, there is no effect of a COVID-19 infection. Again, people living in households in a tighter financial situation are somewhat more likely to report more positive change. There are no differences between men and women, and among regions. While civic engagement prior to the pandemic has no effect, more religious people experienced more positive and thus less negative change in generalized trust. Regarding the additional control variables that are not reported in Table 2, older people generally report less negative changes, although this effect is inversely U-shaped. There are no differences in the change of trust during the pandemic regarding people’s education, occupational status, residential mobility, partnership status, neighborhood vandalism, migration history, or whether they have children.

### 4.3 Robustness

As a first test, the models in Table 1, Table 2 are rerun on the sample of people who did not move house between 2019 and 2020, since they likely evaluate their neighborhood network differently from those who stayed. Removing the 4.5% of movers from the sample, however, results in the same pattern of effects in all the analyses.

On another account, Table 1 demonstrates that the change in neighboring is endogenous. If the covariates in Table 2 do not account for this endogeneity, results are likely biased (Wooldridge, 2010). As a second test, we thus check whether the change in neighboring can be treated as exogenous in the second part of the analyses. To do so, the residuals of a regression of the change score on all the covariates are included in the second step of analysis (Durbin–Wu–Hausman test).

The test-statistics in Table 3 are based on 500 bootstrap samples. While we clearly fail to reject the null-hypothesis of an exogenous influence when modeling the change in subjective well-being (*χ*^2^ (1) = 0.063, *p* = 0.802), the situation is less clear in the case of generalized trust. The *χ*^2^ value of 3.177 and the corresponding *p*-value of 0.075 suggest that the effect of improving neighborly relations on the change in generalized trust during COVID-19 could be endogenous and calls for a more cautious interpretation of the results.

**Table 3:** Testing endogeneity.

	500 bootstrapped samples	
	*χ*^2^ (1)	*p*−value
Change score subjective well-being	0.063	0.802
Change score generalized trust	3.177	0.075

As a third, more general test of the models presented thus far, a placebo regression approach is followed. It was argued that social distancing measures and stay-at-home orders made localized social capital to a primary source of help and contact during the COVID-19 crisis. Compared to pre-pandemic times, changes in neighborly relations should thus be of special importance for people’s subjective well-being and trust. Consequently, applying the models to the pre-crisis time should not result in the documented strong association with changes in neighborly relations. Since people’s networks are only inquired every third year, the models are re-estimated for the time between 2016 and 2019. However, the change in neighborly relations has to be constructed. To this end, the difference in self-reported frequency of contact with neighbors is used. Consequently, it cannot be ruled out that differences to the main analyses occur due to differences in measurement. Other network variables (size of one’s neighborhood, friendship, and online social network) are assessed by means of the 2016 measures. All the other variables are the same as in the main analyses.

Table 4 shows that the change in neighborly relations between 2016 and 2019 has no significant influence on the change in subjective well-being and trust in other people during that period. Other network measures have only a minor influence. All the other model variables are included but not reported. They are mostly insignificant (see Table A2 in the appendix for the full results). The absence of an effect of changes in neighborly relations makes us more confident that the pattern found in the main analyses can be attributed to the unique circumstances of the pandemic.

**Table 4:** Placebo regression: changes between 2019 and 2016.

	2019 vs. 2016			
	Change score SWB		Change score trust	
Change neighborly relations 2019 vs. 2016	0.009	(0.016)	0.042	(0.022)
(Change neighborly relations)^2^ 2019 vs. 2016	-0.005	(0.006)	-0.014	(0.008)
Log(size neighborhood network) 2016	-0.003	(0.004)	0.000	(0.006)
Log(size friendship network) 2016	0.005	(0.007)	0.021*	(0.010)
Log(size online social network) 2016	0.006*	(0.003)	-0.001	(0.004)
Observations	4177		4164	
*R*^2^	0.017		0.013	

Standard errors in parentheses; Controlled for all the covariates in the original models. *p < 0.05, **p < 0.01, ***p < 0.001.

## 5 Discussion

This paper pursued two goals. First, using panel data from Switzerland, it investigated for whom relations with neighbors changed during the COVID-19 pandemic. Second, and more importantly, the paper made a first and internationally unique assessment of how improving neighborly relations buffered the negative effects of the pandemic on people’s subjective well-being and trust. Against the background of the importance of neighbors in times of crisis (Aldrich & Meyer, 2015; LaLone, 2012), it was argued that neighbors and localized social capital were especially important during the pandemic since help and social contacts had to be organized locally due to social distancing and stay-at-home orders.

In line with a previous study that found a buffering effect of neighborhood infrastructure on people’s mental health during COVID-19 (Miao et al., 2021), this study demonstrated that an improvement in one’s relation with neighbors significantly reduced the negative impact of the crisis on people’s subjective well-being and trust, although in a nonlinear fashion. This finding is also in line with work that more generally stresses the importance of perceived changes for individual subjective well-being (Greenfield & Reyes, 2015). In magnitude, the documented effects are comparable to the one of individual subjective health and are considerably stronger than other network influences.

However, not everybody was equally likely to improve their relations with neighbors during the pandemic. In line with evidence from Sweden (Zetterberg et al., 2021), people who were embedded more strongly in neighborhood, friendship, and online networks prior to the pandemic were more likely to improve relations with their neighbors. Moreover, people who had more resources to cope with the crisis (in terms of health and social status) were more likely to witness improving neighborly relations. Consequently, their subjective well-being and trust was less impacted by the pandemic. In this regard, the socially unequal impact of the COVID-19 pandemic on people’s subjective well-being and trust (Zacher & Rudolph, 2021) partly also works through changes induced in people’s neighborhood social network.

### 5.1 Limitations

There are several aspects to keep in mind regarding the presented findings. First, while this study broadened the scope of existing research (Miao et al., 2021; Zetterberg et al., 2021) by explicitly focusing on neighborhood ties rather than infrastructure, and covered not only urban but also suburban and rural settings, the point in time and the specific national context limit the generalizability of the results. The first lockdown in Switzerland was not as strict (there were, for example, no curfews) and lasted only 41 days. Consequently, compared to other countries, the impact on people’s subjective well-being might have been less pronounced. Likewise, the time period between the two instances of data collection is quite short and does not allow an assessment of long-lasting impacts of neighboring during COVID-19. Meanwhile, the negative impact of the crisis on people’s trust was found to be enduring and thus might also relate more generally to other contexts.

Second, this study cannot use the full potential of longitudinal data to assess causal effects. Changes in neighborly relations during the pandemic reflect a subjective assessment. The change score of neighborly relations in this study thus differs methodologically from “traditional” change score analysis (Allison, 1990; Morgan & Winship, 2014). Meanwhile, this is not the case when analyzing how improving relations with neighbors buffered the negative impact of the pandemic on people’s subjective well-being and trust, since the dependent variables are constructed by differing the values of subsequent waves. Moreover, the placebo regressions, applying the models to pre-COVID-19 waves of the data, reinforce the interpretation of a pandemic-induced change in neighborly relations and their buffering effect on subjective well-being and trust. Nevertheless, reverse causality cannot be ruled out completely: people might have improved their relations with neighbors because they were less affected by the pandemic in terms of subjective well-being and trust. Moreover, such placebo tests are problematic in the sense that it cannot be determined whether the changes in statistical significance are significant themselves (Gelman & Stern, 2006).

Finally, although testing for endogeneity showed that the change in neighborly relations during the pandemic had an exogenous effect on people’s subjective well-being, the result for respondents’ trust in other people, the second dependent variable, was more ambiguous. Against the outlined dependence of changes in neighborly relations on people’s pre-pandemic socio-economic endowments, the results might thus overestimate the impact of the neighborhood network on people’s generalized trust.

### 5.2 Conclusion

Neighbors are an important source of everyday help and support (Greenfield, 2016; LaLone, 2012; Zetterberg et al., 2021). This is especially true in times of crisis, such as the COVID-19 pandemic, when social distancing and stay-at-home orders limit people’s scope of action, contacts and support to the local context.

The results of this study come with several implications for research and policy. First, this study stressed the importance of investigating neighborhood effects not only on objective but also on subjective measures of well-being (Ludwig et al., 2012; Tampubolon, 2012). Second, not everybody was equally likely to improve relations with neighbors during the crisis: people with more resources in terms of education, health, and social networks – as well as women – were more likely to report an increase in neighboring. Consequently, future research as well as public policy should carefully consider the socially unequal impact of initiatives that aim at promoting neighborhood social capital. Third, supporting community resilience can be a powerful approach to help people to get through times of crisis when stay-at-home orders and lockdowns restrict people’s scope of action to the vicinity of their home. In the present case, the effects of improving neighborly relations outweigh many of the individual-level factors. Targeted neighborhood initiatives might therefore not only provide everyday support to neighbors but they are especially suited to protect against the negative impacts of social isolation. Finally, such neighborhood initiatives bear the potential to strengthen social cohesion beyond their local scope. Consequently, local interventions that foster contact, exchange, and interactions among neighbors are of value for the functioning of the whole society.

## Financial disclosure statement

There was no funding for this research. Christoph Zangger is supported by a 10.13039/501100001711SNSF Ambizione Grant by the 10.13039/100000001Swiss National Science Foundation.

## Ethical statement

The paper uses secondary data from the Swiss Household Panel (SHP). Data collection and preparation was completed by the Swiss Centre of Expertise in the Social Sciences (FORS) and followed appropriate ethical procedures.

## Declaration of competing interest

The author declares no conflict of interest.

## Figures and Tables

**Table A1:** Descriptive statistics

	mean	sd	min	max
**Dependent variables**				
Change relation with neighbors 2020 vs. 2019 (categorized)	0.207	0.490	−1.000	1.000
Change life satisfaction 2020 vs. 2019	−0.109	1.360	−10.000	8.000
Change trust 2020 vs. 2019	−0.596	2.052	−10.000	10.000
**Independent variables**				
Change relation with neighbors 2020 vs. 2019	5.439	1.189	0.000	10.000
log(size neighborhood network) 2019	−2.562	5.735	−11.513	2.708
log(size friendship network) 2019	0.876	2.636	−11.513	2.708
log(size online network) 2019	−4.262	8.154	−11.513	6.908
Practical support from neighbors 2019	1.506	1.126	0.000	3.000
Frequency of contact with friends 2019	2.485	0.762	0.000	3.000
Has partner				
No	0.200	0.400	0.000	1.000
Yes, in same household	0.709	0.454	0.000	1.000
Yes, in another household	0.090	0.286	0.000	1.000
Has children				
No	0.781	0.413	0.000	1.000
Yes	0.219	0.413	0.000	1.000
Voluntary work in 2019	0.393	0.488	0.000	1.000

Household moved since last wave	0.047	0.213	0.000	1.000
Days since the end of lockdown	26.652	11.796	16.000	64.000
COVID-19 infection				
No	0.539	0.499	0.000	1.000
Respondent or someone in HH	0.017	0.129	0.000	1.000
Someone respondent knows personally	0.444	0.497	0.000	1.000
Subjective health	4.308	0.672	1.000	5.000
Labor market status				
Employed	0.498	0.500	0.000	1.000
Self-employed	0.066	0.249	0.000	1.000
Inactive	0.436	0.496	0.000	1.000
Age	55.666	17.209	18.000	99.000
Household financial situation				
HH can save money	0.598	0.490	0.000	1.000
HH spends what it earns	0.301	0.459	0.000	1.000
HH eats its assets or gets into debt	0.101	0.301	0.000	1.000
Highest completed education				
At most compulsory (ISCED 2)	0.084	0.278	0.000	1.000
Upper secondary, non-tertiary (ISCED 3–4)	0.595	0.491	0.000	1.000
Tertiary (ISCED 5–8)	0.321	0.467	0.000	1.000

Neighborhood vandalism	0.047	0.213	0.000	1.000
Gender				
Man	0.458	0.498	0.000	1.000
Woman	0.542	0.498	0.000	1.000
Duration residence in Switzerland				
Since birth	0.768	0.422	0.000	1.000
Up to 10 years	0.010	0.098	0.000	1.000
More than 10 years	0.222	0.416	0.000	1.000
Religiosity	2.326	1.147	1.000	5.000
Community typology				
Centers	0.100	0.300	0.000	1.000
Suburban communes	0.204	0.403	0.000	1.000
Wealthy communes	0.062	0.241	0.000	1.000
Peripheral urban communes	0.148	0.355	0.000	1.000
Tourist communes	0.030	0.171	0.000	1.000
Industrial and tertiary sector communes	0.116	0.320	0.000	1.000
Rural commuter communes	0.181	0.385	0.000	1.000
Mixed agricultural communes	0.114	0.318	0.000	1.000
Peripheral agricultural communes	0.046	0.209	0.000	1.000
*N*	4530			

**Table A2:** Placebo regressions, full results

	2019 vs. 2016			
	Change score SWB		Change score trust	
Change neighborly relations 2019 vs. 2016	0.009	(0.016)	0.042	(0.022)
(Change neighborly relations 2019 vs. 2016)^2^	−0.005	(0.006)	−0.014	(0.008)
Log(size neighborhood network) 2016	−0.003	(0.004)	0.000	(0.006)
Log(size friendship network) 2016	0.005	(0.007)	0.021*	(0.010)
Log(size online social network) 2016	0.006*	(0.003)	−0.001	(0.004)
Days since end of lockdown	−0.002	(0.002)	−0.001	(0.002)
Health	0.077**	(0.028)	−0.001	(0.039)
COVID-19 infection				
respondent or someone in household	−0.023	(0.146)	−0.081	(0.204)
someone respondent knows personally	−0.026	(0.040)	0.037	(0.056)
Age	0.022**	(0.007)	0.004	(0.010)
Age^2^	−0.000*	(0.000)	−0.000	(0.000)
Household finances				
HH spends what it earns	−0.028	(0.044)	−0.239***	(0.061)
HH eats its assets or gets into debt	−0.141*	(0.067)	−0.219*	(0.093)
Education				
upper secondary schooling	−0.027	(0.072)	0.135	(0.100)
tertiary schooling	−0.047	(0.078)	0.027	(0.109)

Gender				
woman	0.039	(0.039)	−0.017	(0.055)
Feeling of religiosity	0.017	(0.017)	−0.021	(0.024)
Community typology				
suburban communes	0.040	(0.073)	0.079	(0.102)
wealthy communes	0.074	(0.097)	0.252	(0.136)
peripheral urban communes	0.034	(0.078)	0.104	(0.109)
tourist communes	0.135	(0.123)	0.138	(0.173)
industrial and tertiary sector communes	−0.007	(0.083)	0.016	(0.116)
rural commuter communes	−0.035	(0.075)	0.083	(0.105)
mixed agricultural communes	−0.038	(0.083)	0.115	(0.116)
peripheral agricultural communes	0.061	(0.107)	0.199	(0.150)
Partner				
yes, in same household	0.073	(0.052)	0.087	(0.072)
yes, in another household	0.129	(0.077)	0.072	(0.109)
Children in HH	−0.094	(0.052)	0.044	(0.073)
Occupational status				
self-employed	0.119	(0.079)	0.124	(0.110)
inactive	−0.027	(0.054)	0.023	(0.076)
Duration residence in CH				
up to 10 years	−0.491*	(0.201)	−0.041	(0.281)
more than 10 years	0.042	(0.046)	0.031	(0.064)
Voluntary work	0.028	(0.040)	0.041	(0.056)
Problems with vandalism in neighborhood	−0.064	(0.090)	−0.018	(0.126)
Household moved since last wave	0.113	(0.103)	0.136	(0.144)
Observations	4′177		4′164	
*R*^2^	0.017		0.013	

Standard errors in parentheses.

*p < 0.05, **p < 0.01, ***p < 0.001.

## Data Availability

The data is available on swissubase.ch
